# The PPAR**γ** Agonist Rosiglitazone Is Antifibrotic for Scleroderma Lung Fibroblasts: Mechanisms of Action and Differential Racial Effects

**DOI:** 10.1155/2012/545172

**Published:** 2011-11-01

**Authors:** Galina S. Bogatkevich, Kristin B. Highland, Tanjina Akter, Richard M. Silver

**Affiliations:** Division of Rheumatology and Immunology, Medical University of South Carolina, 96 Jonathan Lucas Street, Suite 912, Charleston, SC 29425-6370, USA

## Abstract

We present novel data demonstrating that the expression of PPAR**γ** is reduced in lung fibroblasts from black SSc-ILD patients as compared to white patients. Activating PPAR**γ** with the agonist rosiglitazone increased the expression of MMP-1 and inhibited collagen type I in lung fibroblasts isolated from white, but not black, SSc-ILD patients. Blocking the c-Met receptor abolishes rosiglitazone's effects on collagen and MMP-1 in lung fibroblasts isolated from white SSc-ILD patients, while augmenting the expression of the c-Met receptor in fibroblasts from black SSc-ILD patients replicates the effects of rosiglitazone seen in whites. We conclude that PPAR**γ** agonists warrant consideration as potential antifibrotic drugs in patients with SSc-ILD. Differential therapeutic effects might be anticipated especially relative to racial differences and the functional expression of the c-Met receptor.

## 1. Introduction

PPAR*γ* is a ligand-dependent transcription factor belonging to the nuclear steroid/retinoid/vitamin D receptor superfamily that plays a pivotal role in the regulation of adipogenesis, insulin sensitivity, glucose homeostasis, and immune response (reviewed in [1, 2]). Activation of PPAR*γ* inhibits the proinflammatory effects of lipopolysaccharide and various cytokines on immune cells. PPAR*γ* is detectable in normal lung where it is expressed in epithelial cells, smooth muscle cells, and alveolar macrophages [3–5]. Reduced PPAR*γ* nuclear protein and gene expression has been demonstrated in dermal fibroblasts and in lung and skin biopsies from patients with SSc [6, 7], and in alveolar macrophages of patients with sarcoidosis and pulmonary alveolar proteinosis [4, 8], suggesting that insufficient PPAR*γ* activity may contribute to ongoing dysregulated inflammation and fibrosis. 

In normal skin fibroblasts, ligand activation of cellular PPAR*γ* has been shown to reduce basal collagen gene expression and abrogate TGF-*β*-induced stimulation [9]. PPAR*γ* ligands also abrogate TGF-*β*-induced expression of *α*-SMA, a marker of myofibroblasts, and suppress stimulation of Smad-dependent transcriptional responses to TGF-*β* [9]. Recently, Kapoor et al. demonstrated that a loss of PPAR*γ* in mouse fibroblasts results in increased susceptibility to bleomycin-induced skin fibrosis [10]. Activating PPAR*γ* with rosiglitazone was shown to alleviate the persistent fibrotic phenotype of lesional skin scleroderma fibroblasts [6] and to attenuate inflammation, dermal fibrosis, and subcutaneous lipoatrophy in a murine model of scleroderma [11], suggesting that PPAR*γ* ligands may be considered as potential therapeutic agents for scleroderma. 

 Similar effects have been reported from studies of lung fibroblasts [12]. Burgess et al. have shown that both endogenous and synthetic PPAR*γ* agonists (15d-PGJ_2_ and ciglitazone or rosiglitazone, resp.) are able to block key TGF-*β*-mediated profibrotic effects *in vitro*, including pulmonary myofibroblast differentiation and excess collagen production, without affecting cell viability. 15d-PGJ_2_ inhibited >95% of the TGF-*β*-stimulated *α*-SMA induction, whereas rosiglitazone inhibited 40% of the TGF-*β*-stimulated *α*-SMA [12]. Such findings may be particularly relevant to SSc-ILD, since we and others have demonstrated elevated levels of TGF-*β* [13, 14], as well as the presence of myofibroblasts expressing increased levels of *α*-SMA [15] in bronchoalveolar lavage fluid (BALF). The present study demonstrates *in vitro* antifibrotic effects of the PPAR*γ* agonist rosiglitazone in lung fibroblasts derived from patients with SSc-ILD, providing additional support for a potential new role for PPAR*γ* agonists as antifibrotic therapy in patients with SSc-ILD.

## 2. Materials and Methods

### 2.1. Materials

Rosiglitazone and GW 9662 were purchased from Cayman Chemical, Ann Arbor, Mich, USA; EnzoLytePlus 520 MMP-1 Assay Kit was obtained from AnaSpec (San Jose, Calif, USA); NoShift Transcription Factor Assay Kit, NoShift NF-*κ*B (p65) reagents, and NucBuster Protein Extraction Kit were purchased from Novagen (Madison, Wis); MMP inhibitor GM1489 was obtained from EMD Biosciences (San Diego, Calif). Anti-PPAR*γ* polyclonal antibody was purchased from Cell Signaling Technology (Danvers, Mass); anti-*α*-SMA and anti-*β*-actin antibodies were purchased from Sigma-Aldrich (St. Louis, Mo); antitype I collagen antibody was purchased from SouthernBiotech (Birmingham, Alaska). Anti-CTGF antibody and anti-Met (C-12) antibody were obtained from Santa Cruz Biotechnology (Santa Cruz, Calif). c-Met-pLXSN cDNA was kindly provided by Dr. Morag Park (McGill University, Montreal, Canada). Rosiglitazone was prepared as 20 mM, GW 9662 as 2 mM, and GM1489 as 1 mM stocks in DMSO, and the same amounts of DMSO as in rosiglitazone-, GW 9662-, and GM1489-treated cells were added to control cell cultures. 

### 2.2. Cell Culture and Transfection

Lung fibroblasts were derived from lung tissue obtained at autopsy from 5 White and 5 African American patients with end-stage SSc lung disease (1 male, 4 females per each group) and from normal subjects (1 male, 2 females per each group) as described previously [16]. Lung fibroblasts were used between the second and fourth passages in all experiments. The mean ± SD age of the SSc patients was 57.8 ± 10.2 years in the white group and 42.2 ± 9.7 in the African American group. The mean ± SD age of normal subjects was 53.3 ± 4.7 years in the whites and 46.3 ± 5.6 in the African American group. The difference in age between whites and African Americans was not statistically significant either in control or in scleroderma patients (*P* > 0.05). In one set of experiments, cells were transfected with c-Met-pLXSN cDNA by Effectene reagent (Qiagen, Valencia, Calif) according to manufacturer's instructions as described previously [17].

### 2.3. Enzyme-Linked Immunosorbent Assay (ELISA) for Hepatocyte Growth Factor (HGF)

Levels of HGF were measured in 50 *μ*L samples of cell culture medium using Quantikine human HGF ELISA kit (R & D Systems, Minneapolis, Minn) according to manufacturer's instructions as described previously [16]. 

### 2.4. EnzoLytePlus Assay

MMP-1 activity was measured in samples consisting of 100 *μ*L of cell culture medium by EnzoLytePlus 520 Assay according to the manufacturer's instructions as described previously [18]. 

### 2.5. NF-*κ*B DNA-Binding Activity Assay

Nuclear protein extracts were prepared from lung fibroblasts incubated with and without rosiglitazone (20 *μ*M) for 24 hours using NucBuster Protein Extraction Kit from Novagen in accordance with manufacturer's instructions. Protein concentration was determined using BCA protein assay kit (Pierce, Rockford, Ill). Nuclear extracts (25 *μ*g each) from lung fibroblasts or from HeLa-positive control cells provided by manufacturer were incubated with various combinations of biotinylated NF-*κ*B wild-type dsDNA, specific NF-*κ*B competitor dsDNA lacking biotin end labels, and nonspecific, nonbiotinylated dsDNA with a mutant NF-*κ*B consensus binding motif. DNA-binding activity of NF-*κ*B was determined using the NoShift Transcription Factor Assay Kit and NoShift NF-*κ*B (p65) reagents as described previously [18].

### 2.6. Preparation of Cell Extracts and Immunoblotting

Lung fibroblasts were cultured to confluence on 100 mm dishes, maintained in serum-free DMEM overnight, and then treated with or without rosiglitazone. In some experiments, cells were pretreated with neutralizing anti-c-Met antibody (R & D Systems, Minneapolis, Minn), PPAR*γ* antagonist GW 9662 or MMP inhibitor GM1489. Normal goat IgG served as a negative control with c-Met neutralizing antibody. Lung fibroblasts were washed with ice-cold PBS and lysed with ice-cold lysis buffer (10 mM Tris, 10 mM EDTA, 1% Nonidet P-40, 0.5% deoxycholate, 0.1% SDS, pH = 7.4). Protein concentration was determined by BCA protein assay in accordance with manufacturer's instructions (Pierce, Rockford, Ill). For each sample, 40 *μ*g of protein was denatured, subjected to SDS-polyacrylamide gel electrophoresis, and analyzed by immunoblotting with appropriate antibodies. The immunoblots were then stripped and reblotted with anti-*β*-actin antibody as a loading control. Western blotting for CTGF was performed as described previously [19].

### 2.7. c-Met Receptor Phosphorylation

Scleroderma lung fibroblasts were cultured to confluence on six-well plates, maintained in serum-free DMEM overnight, and then treated with or without rosiglitazone for various times. The cells were transferred on ice, washed with ice-cold PBS, collected with 1 × SDS sample buffer (100 *μ*L/well) for denaturing gel electrophoresis (4–20% SDS-PAGE), and immunoblotted with anti-phospho-c-Met [pYpYpY^1230/1234/1235^] polyclonal antibody (BioSource International, Inc., Camarillo, Calif). In each experiment, the membrane transfers used for immunoblotting were evaluated for total amount of c-Met receptor by reblotting with anti-Met (C-12) polyclonal antibody (Santa Cruz Biotechnology). 

### 2.8. Statistical Analysis

Statistical analyses were performed with KaleidaGraph 4.0 (Synergy Software, Reading, Pa). All data were analyzed using nonparametric Wilcoxon-Mann-Whitney tests and ANOVA with Tukey HSD post hoc testing. The results were considered significant if *P* < 0.05. 

## 3. Results

### 3.1. PPAR*γ* Expression in Lung Fibroblasts

**Figure 1:**
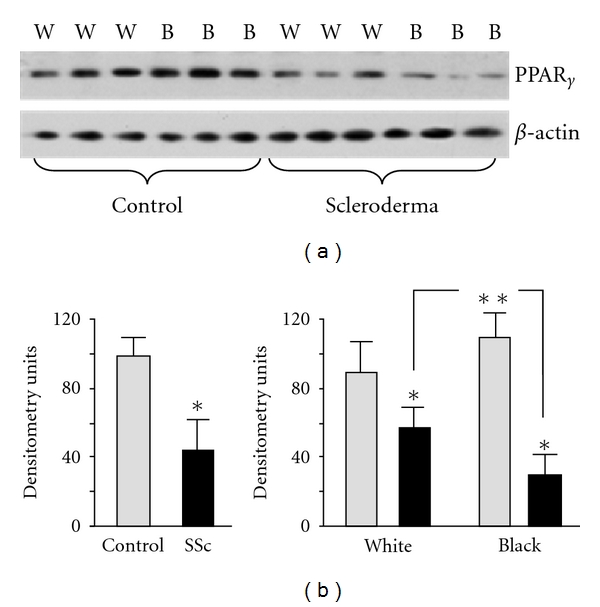
PPAR*γ* expression in lung fibroblasts. (a) Western blot for PPAR*γ* on lung fibroblasts isolated from white (W) and black (B) SSc-ILD patients and controls. Anti-*β*-actin antibody was used as a loading control. (b) Densitometric analysis of PPAR*γ* in lung fibroblasts (*n* = 16) from 3 independent experiments is presented. PPAR*γ* protein expression was adjusted in accordance with *β*-actin level. *Statistically significant differences.

To better understand the role of PPAR*γ* in the pathogenesis of SSc lung disease, we investigated the expression of PPAR*γ* in lung fibroblasts isolated from SSc-ILD patients and controls. We measured PPAR*γ* expression by Western blotting in 6 controls (3 white and 3 black) and 10 SSc fibroblast cell lines isolated from patients with end-stage SSc-ILD (5 white and 5 black). PPAR*γ* levels were significantly reduced in SSc lung fibroblasts (43.5 ± 18.3 versus 101.1 ± 7.9, *P* < 0.001) (Figure 1). We next compared PPAR*γ* expression between fibroblasts derived from white and black normal controls and SSc patients. We found that lung fibroblasts from white SSc-ILD patients had significantly greater PPAR-*γ* expression compared to black SSc-ILD fibroblasts (57.4 ± 11.8 versus 29.6 ± 11.3, *P* < 0.05).

### 3.2. Differential Effects of PPAR*γ* in Fibroblasts from White and Black SSc-ILD Patients

**Figure 2:**
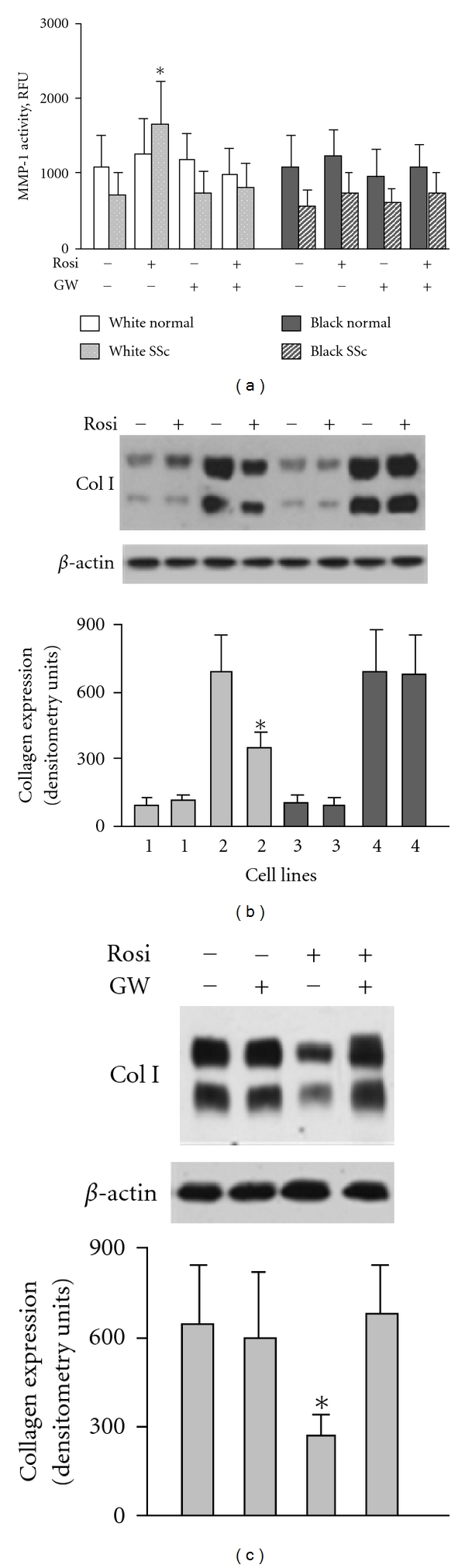
Effect of rosiglitazone on MMP-1 and collagen I in lung fibroblasts. (a) MMP-1 activity in lung fibroblasts. Each bar represents the mean and SD of duplicate determinations in 4 independent experiments. (b) Effect of rosiglitazone on collagen type I in white normal (1), white scleroderma (2), black normal (3), and black scleroderma (4) lung fibroblasts. (c) PPAR*γ* antagonist GW inhibits effect of rosiglitazone on collagen in white scleroderma fibroblasts. Immunoblots in (b) and (c) are representative of 5 independent experiments. The results of immunoblot analysis were quantified. Values are the mean and SD of densitometry units corresponding to the *α*1 and *α*2 chains of type I collagen. *P < 0.05 versus unstimulated cells.

We sought to compare the effects of rosiglitazone in different lines of lung fibroblasts. We tested 10 SSc-ILD cell lines (5 from black and 5 from white patients) and 6 normal lung fibroblasts (3 from black and 3 from white). When lung fibroblasts were treated with rosiglitazone in a concentration of 20 *μ*M for 24 hours, MMP-1 activity was increased in lung fibroblasts from white SSc-ILD patients; such treatment, however, had no effect on MMP-1 activity in SSc-ILD fibroblasts from black patients (Figure 2(a)). Rosiglitazone notably reduced collagen after 48 hours of treatment in lung fibroblasts from white but not black SSc-ILD patients (Figure 2(b)). The effects of rosiglitazone on MMP-1 and collagen were inhibited by the PRAR*γ* antagonist, GW 9662, indicating a PPAR*γ*-dependent mechanism (Figures 2(a) and 2(c)). Rosiglitazone had no effects on MMP-1 and collagen in either white or black normal lung fibroblasts.

### 3.3. Effect of Rosiglitazone on PPAR*γ* Expression, CTGF, *α*-SMA, and NF-*κ*B DNA Binding Activity in Lung Fibroblasts

**Figure 3:**
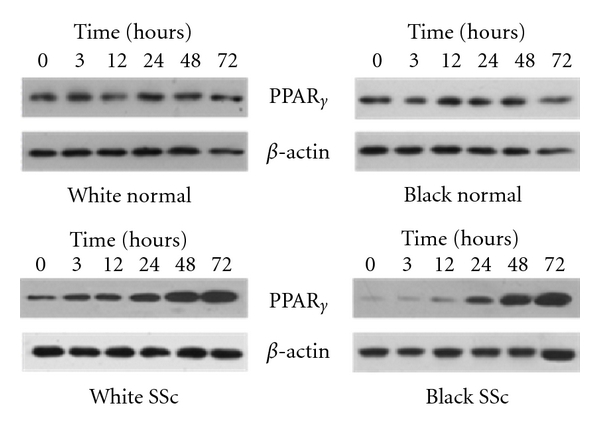
Rosiglitazone increases PPAR*γ* expression in SSc lung fibroblasts in a time-dependant manner. Lung fibroblasts were incubated with rosiglitazone (20 *μ*M) for time indicated. Cells were collected and subjected to Western analysis for PPAR*γ* expression. Anti-*β*-actin antibody was used as a loading control. Immunoblots are representative from 3 independent experiments.

To investigate whether rosiglitazone would increase the level of PPAR*γ* in lung fibroblasts, we incubated cells with rosiglitazone for different times and then analyzed PPAR*γ* expression by immunoblotting. We observed that exposure of fibroblasts to rosiglitazone for ≥12 hours results in significantly increased levels of PPAR*γ* in both white and black SSc lung fibroblasts. However, rosiglitazone had no effects on PPAR*γ* expression in normal lung fibroblasts (Figure 3). 

**Figure 4:**
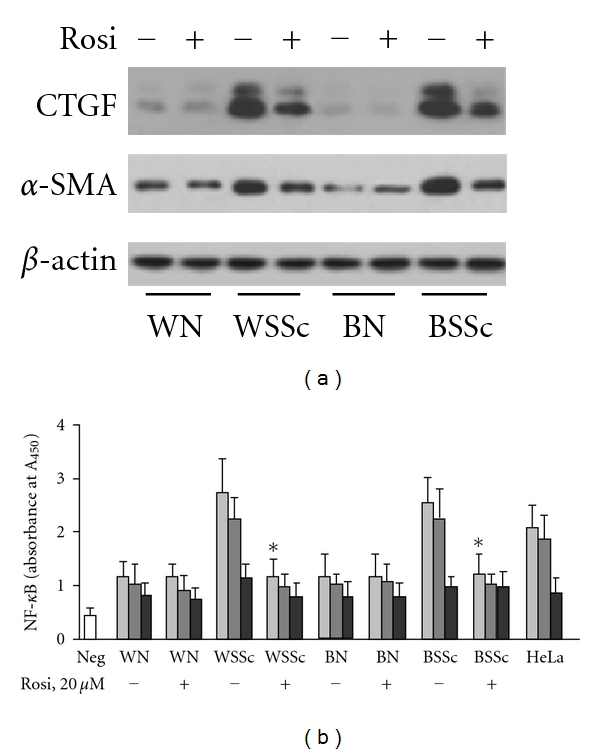
Effect of rosiglitazone on CTGF, *α*-SMA, and NF-*κ*B in lung fibroblasts isolated from white (W) and black (B) SSc-ILD patients (SSc) and controls (N). (a) Lung fibroblasts were incubated with rosiglitazone (20 *μ*M) for 24 hours and subjected to Western analysis for CTGF and *α*-SMA expression. Anti-*β*-actin antibody was used as a loading control. Immunoblots are representative from 5 independent experiments. (b) NF-*κ*B DNA-binding activity. Light gray bars: binding activity assessed by biotinylated NF-*κ*B wild-type (WT) double-stranded DNA (dsDNA); dark gray bars: WT dsDNA plus nonspecific, nonbiotinylated dsDNA with a mutant NF-*κ*B consensus-binding motif; black bars: WT dsDNA plus specific NF-*κ*B competitor dsDNA lacking biotin end labels; Neg: negative control. Each bar represents the mean ± SD of duplicate determinations from 4 independent experiments. *Statistically significant differences between nuclear extract prepared from cells stimulated with rosiglitazone versus nuclear extracts from nonstimulated cells (*P* < 0.05).

 Rosiglitazone has been reported to suppress CTGF and *α*-SMA protein expression in lesional SSc skin fibroblasts [6]. To determine whether rosiglitazone inhibits expression of CTGF and *α*-SMA in various SSc lung fibroblasts, we investigated protein expression of CTGF and *α*-SMA with and without rosiglitazone in all tested cell lines. We found that rosiglitazone reduces CTGF and *α*-SMA expression in all SSc cell lines within 24 hours of treatment. No difference between white and black lung fibroblasts was observed. Also, rosiglitazone had no effects on CTGF and *α*-SMA in normal lung fibroblasts (Figure 4(a)).

 Since PPAR*γ* agonists are known to inhibit NF-*κ*B [20], we examined the effect of rosiglitazone on NF-*κ*B activation in lung fibroblasts. NF-*κ*B DNA-binding activity was assayed by the binding of NF-*κ*B to oligonucleotides containing the consensus binding site. Nuclear extracts from HeLa cells stimulated with TNF-*α* were used as a positive control. To assess sequence-specific binding activity, nuclear extracts were incubated with NF-*κ*B wild-type DNA, with or without either a specific NF-*κ*B competitor DNA or nonspecific mutant NF-*κ*B consensus-binding motif. Nuclear extracts incubated with NF-*κ*B wild-type DNA expressed actual NF-*κ*B activity. The specific NF-*κ*B competitor DNA reduced the binding activity in all cell lines confirming sequence specificity of the assay for NF-*κ*B binding, but binding activity of a nonspecific mutant did not significantly differ among various conditions used in this experiment (Figure 4(b)). Consistent with our previous observations [18], basal levels of active NF-*κ*B in SSc-ILD fibroblasts were higher than in TNF-*α*-induced HeLa cells used as a positive control. Nuclear extracts prepared from either white or black SSc-ILD fibroblasts treated with rosiglitazone demonstrated significant reduction of NF-*κ*B DNA binding (Figure 4(b)). NF-*κ*B DNA binding activity of normal lung fibroblasts was notably reduced as compared with binding activity of SSc lung fibroblasts and was not affected by rosiglitazone (Figure 4(b)).

### 3.4. The Effects of Rosiglitazone on HGF and c-Met in Lung Fibroblasts Isolated from SSc-ILD Patients

**Figure 5:**
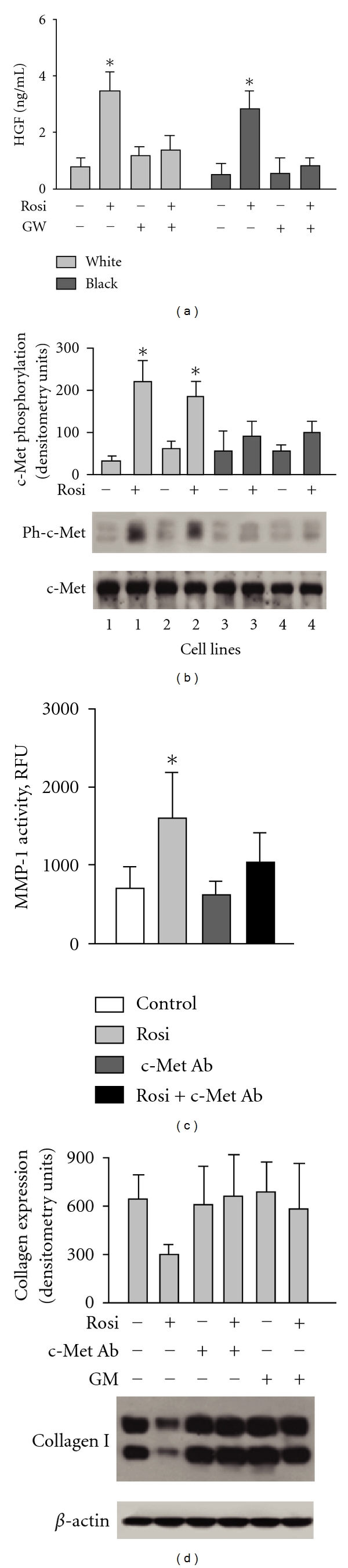
Effects of rosiglitazone in lung fibroblasts are mediated in part by HGF. (a) Rosiglitazone induces HGF expression in SSc lung fibroblasts. Each bar represents the mean ± SD of duplicate determinations in four independent experiments. (b) Effect of rosiglitazone on c-Met receptor phosphorylation in lung fibroblasts from white (cell lines 1 and 2) and black (cell lines 3 and 4) patients with SSc-ILD. (c) Anti-c-Met antibody modulates MMP-1 activity in white SSc lung fibroblasts. (d) Downregulation of collagen type I by rosiglitazone in white SSc lung fibroblasts depends on c-Met and MMP-1. Quantitative results of densitometric analysis of immunoblots presented in (b) and (d) are shown; values are the mean and SD from 4 independent experiments. *P < 0.05 versus unstimulated cells.

Since PPAR*γ* binds to the peroxisome proliferator response element in the HGF promoter region and induces HGF expression in renal mesangial cells [21], we tested the effects of rosiglitazone on HGF expression in lung fibroblasts. We found that rosiglitazone induced the secretion of HGF protein in cell supernatant to a similar extent in lung fibroblasts from both white and black subjects (Figure 5(a)). Next, we tested whether HGF induced by PPAR*γ* agonist was sufficient to phosphorylate the c-Met receptor in lung fibroblasts. We measured the level of total and phosphorylated c-Met receptor using anti-Met or anti-phospho-c-Met [pYpYpY^1230/1234/1235^] polyclonal antibodies and found that rosiglitazone had no effect on the total amount of c-Met receptor protein, yet it induced c-Met receptor phosphorylation in SSc-ILD lung fibroblasts from white patients (Figure 5(b)). We previously demonstrated that lung fibroblasts from black SSc patients are unresponsive to HGF signaling and c-Met receptor phosphorylation [16]. In agreement with those data, we observed that rosiglitazone did not affect c-Met receptor phosphorylation in lung fibroblasts from black SSc-ILD patients (Figure 5(b)).

### 3.5. HGF Receptor c-Met Mediates the Effects of Rosiglitazone on Collagen in Lung Fibroblasts Isolated from White SSc-ILD Patients

To investigate whether rosiglitazone-induced inhibition of MMP-1 and collagen type I is mediated via a c-Met receptor-dependent mechanism, we employed an anti-human HGF R (c-Met) antibody. This antibody was selected for its ability to neutralize the receptor-ligand interaction. We observed that the pretreatment of lung fibroblasts with neutralizing anti-c-Met antibody (2 *μ*g/mL) prevents rosiglitazone-induced MMP-1 activity and reduces rosiglitazone's inhibitory effect on collagen expression (Figures 5(c) and 5(d)). We also found that the MMP inhibitor, GM1489, decreases the inhibitory effect of rosiglitazone on collagen (Figure 5(d)). 

**Figure 6:**
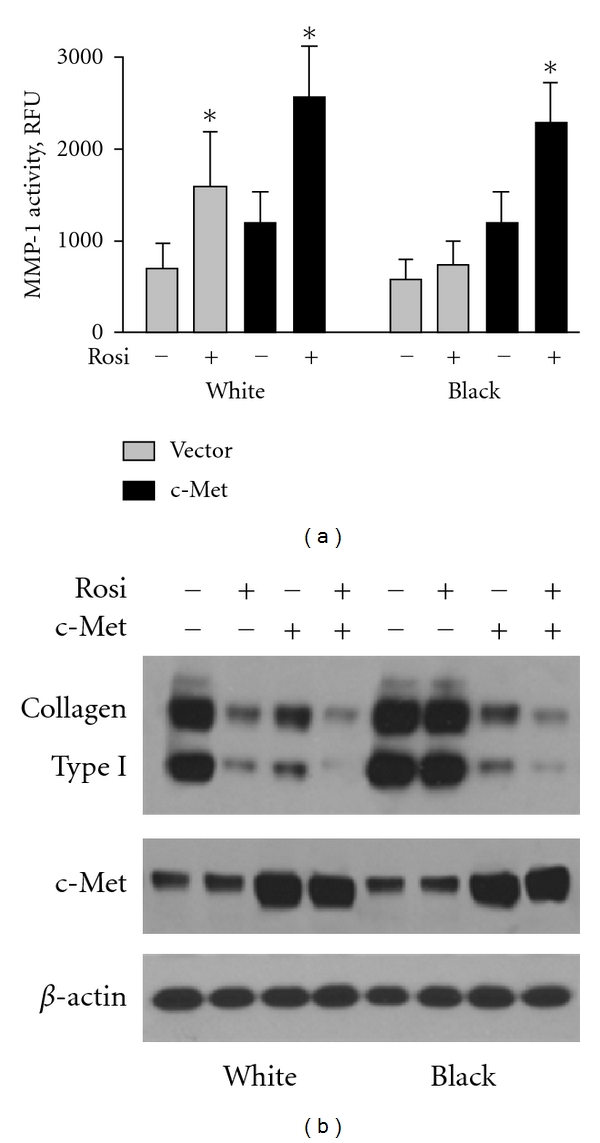
Overexpression of c-Met receptor in lung fibroblasts restores effects of rosiglitazone on MMP-1 (a) and collagen type I (b) in black lung fibroblasts. (a) Each bar represents the mean ± SD of duplicate determinations in four independent experiments. *Statistically significant differences between cells stimulated with rosiglitazone versus nonstimulated cells (*P* < 0.05). (b) Representative immunoblots from 3 independent experiments performed with 5 white and 5 black cell lines are presented. Anti-*β*-actin antibody was used as the sample loading control.

 Overexpression of c-Met in lung fibroblasts isolated from white SSc-ILD patients resulted in increased basal and rosiglitazone-induced activity of MMP-1; however, the difference between vector- and c-Met-transfected cells was not significant (Figure 6(a)). In contrast, we observed significant changes between lung fibroblasts transfected with vector and c-Met in cells isolated from black SSc-ILD patients. Moreover, rosiglitazone significantly increased MMP-1 activity in lung fibroblasts derived from black patients after transfection with c-Met receptor (Figure 6(a)). Overexpression of c-Met in lung fibroblasts isolated from either white or black scleroderma patients results in reduced accumulation of collagen. Treatment of such cells with rosiglitazone further decreased the amount of collagen in these cells (Figure 6(b)).

## 4. Discussion

Interstitial lung disease (ILD) is an irreversible and progressive disease process and the major cause of death among scleroderma patients (SSc-ILD). Characterized by microvascular injury and inflammation, SSc-ILD culminates in excessive deposition of extracellular matrix proteins, often resulting in severe lung dysfunction and death [22, 23]. African-American (black) SSc patients have a higher risk of developing SSc-ILD and tend to develop lung fibrosis earlier in the course of their disease and have a higher mortality rate than white SSc patients [24]. Black SSc patients also have a higher prevalence of the severe diffuse type of SSc and a higher rate of the anti-Scl-70 autoantibody (antitopoisomerase I), which appears to be associated with an increased risk for SSc lung disease with severe pulmonary fibrosis [25–27]. Despite these well-known associations, a potential pathophysiologic link between black race and pulmonary fibrosis in SSc has not yet been identified. We previously reported that compared to white SSc-ILD patients, black SSc-ILD patients are deficient in c-Met receptor expression and generate less of the multifunctional and antifibrotic cytokine, hepatocyte growth factor (HGF) [16]. Since HGF expression is induced by PPAR*γ*, the present study was undertaken to investigate potential antifibrotic effects of a PPAR*γ* agonist in lung fibroblasts isolated from SSc-ILD patients, including both black and white subjects.

 The mechanism by which PPAR*γ* exerts its antifibrotic effects is not yet fully understood. It was reported that PPAR*γ* agonists inhibit TGF-*β*-mediated differentiation of fibroblasts to a myofibroblast phenotype, possibly via inhibition of Smad3 phosphorylation and nuclear translocation [28–31]. PPAR-*γ* ligands also block PDGF-dependent proliferation and prolyl4-hydroxylase mRNA [32] and induce tumor-suppressor phosphatase, tensin homologue deleted on chromosome 10 (PTEN), which *in vitro *has been shown to inhibit *α*-SMA and collagen in lung fibroblasts [33]. Recently, Li et al. demonstrated that PPAR*γ* binds to the peroxisome proliferator response element in the HGF promoter region of renal mesangial cells, thereby inducing HGF mRNA expression and protein secretion [21]. In the present study, we demonstrate that *in vitro* treatment with the PPAR*γ* agonist rosiglitazone results in increased HGF protein occurring in association with phosphorylation of the c-Met receptor tyrosine kinase in lung fibroblasts isolated from white, but not black, SSc-ILD patients. 

 The differences between Caucasian and African-American lung fibroblasts were observed in cells derived from SSc patients but not in controls. This would suggest that other ethnic-specific factors related to scleroderma come into play in SSc-ILD. Recently, Hoshino et al. demonstrated an association of HGF promoter polymorphism with severity of ILD in Japanese patients with systemic sclerosis [34]. We previously showed that HGF's antifibrotic effect is significantly reduced in lung fibroblasts isolated from black subjects [16]. Our results were consistent across different cell lines of SSc-ILD fibroblasts or normal lung fibroblasts stimulated with TGF-*β* [16]. HGF consistently inhibited collagen type I and CTGF accumulation in lung fibroblasts derived from white subjects. However, HGF had no effect on collagen or CTGF expression in lung fibroblasts from black patients, previously shown by us to be the result of a deficiency of HGF-receptor (c-Met) phosphorylation [16, 18]. 

 In the present study, we compared the activation of PPAR*γ* in lung fibroblasts isolated from white and black SSc-ILD patients. As expected, the PPAR*γ* agonist failed to induce c-Met receptor phosphorylation in lung fibroblasts from black SSc-ILD patients and controls. Additionally, we observed that the PPAR*γ* agonist rosiglitazone activated MMP-1 and reduced collagen type I in SSc-ILD fibroblasts derived from white subjects; however, it had no effect on MMP-1 and collagen type I in SSc-ILD fibroblasts from black patients. Furthermore, conditional ablation of the c-Met receptor by neutralizing antibody abolished the inhibitory effect of rosiglitazone on MMP-1 and collagen type I, while overexpression of c-Met receptor restored the effects of rosiglitazone on collagen I and MMP-1 in lung fibroblasts isolated from black SSc-ILD patients. In contrast, *α*-SMA, CTGF, and NF-*κ*B were reduced by rosiglitazone treatment to a similar extent in SSc-ILD fibroblasts derived from either black or white subjects, suggesting that the PPAR*γ* agonist reduces *α*-SMA, CTGF, and NF-*κ*B by a mechanism(s) independent of HGF. 

## 5. Conclusions

We conclude that PPAR*γ* agonists modulate important fibrogenic events in lung fibroblasts and could be considered as a potential therapeutic approach to treating patients with SSc-ILD. We also provide additional foundation for the biologic difference between lung fibroblasts from white and black patients, which might explain the observed differences among these racial groups in terms of severity and mortality from SSc-ILD. Finally, based on these results, differential effects of PPAR*γ* agonist therapy might be expected in patients due in part to differences in expression of functional c-Met receptor. 

## References

[1] Consoli A, Devangelio E (2005). Thiazolidinediones and inflammation. *Lupus*.

[2] Becker J, Delayre-Orthez C, Frossard N, Pons F (2006). Regulation of inflammation by PPARs: a future approach to treat lung inflammatory diseases?. *Fundamental and Clinical Pharmacology*.

[3] Patel HJ, Belvisi MG, Bishop-Bailey D, Yacoub MH, Mitchell JA (2003). Activation of peroxisome proliferator-activated receptors in human airway smooth muscle cells has a superior anti-inflammatory profile to corticosteroids: relevance for chronic obstructive pulmonary disease therapy. *Journal of Immunology*.

[4] Bonfield TL, Farver CF, Barna BP (2003). Peroxisome proliferator-activated receptor-*γ* is deficient in alveolar macrophages from patients with alveolar proteinosis. *American Journal of Respiratory Cell and Molecular Biology*.

[5] Wang ACC, Dai X, Luu B, Conrad DJ (2001). Peroxisome proliferator-activated receptor-*γ* regulates airway epithelial cell activation. *American Journal of Respiratory Cell and Molecular Biology*.

[6] Shi-Wen X, Eastwood M, Stratton RJ, Denton CP, Leask A, Abraham DJ (2010). Rosiglitazone alleviates the persistent fibrotic phenotype of lesional skin scleroderma fibroblasts. *Rheumatology*.

[7] Wei J, Ghosh AK, Sargent JL (2010). PPAR*γ* downregulation by TGF*β* in fibroblast and impaired expression and function in systemic sclerosis: a novel mechanism for progressive fibrogenesis. *PLoS One*.

[8] Culver DA, Barna BP, Raychaudhuri B (2004). Peroxisome proliferator-activated receptor *γ* activity is deficient in alveolar macrophages in pulmonary sarcoidosis. *American Journal of Respiratory Cell and Molecular Biology*.

[9] Ghosh AK, Bhattacharyya S, Lakos G, Chen SJ, Mori Y, Varga J (2004). Disruption of transforming growth factor *β* signaling and profibrotic responses in normal skin fibroblasts by peroxisome proliferator-activated receptor *γ*. *Arthritis & Rheumatism*.

[10] Kapoor M, McCann M, Liu S (2009). Loss of peroxisome proliferator-activated receptor *γ* in mouse fibroblasts results in increased susceptibility to bleomycin-induced skin fibrosis. *Arthritis & Rheumatism*.

[11] Wu M, Melichian DS, Chang E, Warner-Blankenship M, Ghosh AK, Varga J (2009). Rosiglitazone abrogates bleomycin-induced scleroderma and blocks profibrotic responses through peroxisome proliferator-activated receptor-*γ*. *The American Journal of Pathology*.

[12] Burgess HA, Daugherty LE, Thatcher TH (2005). PPAR*γ* agonists inhibit TGF-*β* induced pulmonary myofibroblast differentiation and collagen production: implications for therapy of lung fibrosis. *The American Journal of Physiology*.

[13] Bartram U, Speer CP (2004). The role of transforming growth factor *β* in lung development and disease. *Chest*.

[14] Silver RM, Miller KS, Kinsella MB, Smith EA, Schabel SI (1990). Evaluation and management of scleroderma lung disease using bronchoalveolar lavage. *The American Journal of Medicine*.

[15] Ludwicka A, Trojanowska M, Smith EA (1992). Growth and characterization of fibroblasts obtained from bronchoalveolar lavage of patients with scleroderma. *Journal of Rheumatology*.

[16] Bogatkevich GS, Ludwicka-Bradley A, Highland KB (2007). Impairment of the antifibrotic effect of hepatocyte growth factor in lung fibroblasts from African Americans: possible role in systemic sclerosis. *Arthritis & Rheumatism*.

[17] Bogatkevich GS, Gustilo E, Oates JC (2005). Distinct PKC isoforms mediate cell survival and DNA synthesis in thrombin-induced myofibroblasts. *The American Journal of Physiology*.

[18] Bogatkevich GS, Ludwicka-Bradley A, Highland KB (2007). Down-regulation of collagen and connective tissue growth factor expression with hepatocyte growth factor in lung fibroblasts from white scleroderma patients via two signaling pathways. *Arthritis & Rheumatism*.

[19] Bogatkevich GS, Ludwicka-Bradley A, Singleton CB, Bethard JR, Silver RM (2008). Proteomic analysis of CTGF-activated lung fibroblasts: identification of IQGAP1 as a key player in lung fibroblast migration. *The American Journal of Physiology*.

[20] Zhang J, Fang NY, Gao PJ (2008). Peroxisome proliferator-activated receptor-*γ* agonists attenuate angiotensin II-induced collagen type I expression in adventitial fibroblasts. *Clinical and Experimental Pharmacology and Physiology*.

[21] Li Y, Wen X, Spataro BC, Hu K, Dai C, Liu Y (2006). Hepatocyte growth factor is a downstream effector that mediates the antifibrotic action of peroxisome proliferator-activated receptor-*γ* agonists. *Journal of the American Society of Nephrology*.

[22] Highland KB, Silver RM (2005). Clinical aspects of lung involvement: lessons from idiopathic pulmonary fibrosis and the scleroderma lung study. *Current Rheumatology Reports*.

[23] Ostojic P, Cerinic MM, Silver R, Highland K, Damjanov N (2007). Interstitial lung disease in systemic sclerosis. *Lung*.

[24] Nietert PJ, Mitchell HC, Bolster MB, Shaftman SR, Tilley BC, Silver RM (2006). Racial variation in clinical and immunological manifestations of systemic sclerosis. *Journal of Rheumatology*.

[25] Steen VD (2005). Autoantibodies in systemic sclerosis. *Seminars in Arthritis and Rheumatism*.

[26] Greidinger EL, Flaherty KT, White B, Rosen A, Wigley FM, Wise RA (1998). African-American race and antibodies to topoisomerase I are associated with increased severity of scleroderma lung disease. *Chest*.

[27] McNearney TA, Reveille JD, Fischbach M (2007). Pulmonary involvement in systemic sclerosis: associations with genetic, serologic, sociodemographic, and behavioral factors. *Arthritis & Rheumatism*.

[28] Lakatos HF, Thatcher TH, Kottmann RM, Garcia TM, Phipps RP, Sime PJ (2007). The role of PPARs in lung fibrosis. *PPAR Research*.

[29] Milam JE, Keshamouni VG, Phan SH (2008). PPAR-*γ* agonists inhibit profibrotic phenotypes in human lung fibroblasts and bleomycin-induced pulmonary fibrosis. *The American Journal of Physiology*.

[30] Genovese T, Cuzzocrea S, Di Paola R (2005). Effect of rosiglitazone and 15-deoxy-Δ12,14-prostaglandin J2 on bleomycin-induced lung injury. *European Respiratory Journal*.

[31] Zhao C, Chen W, Yang L, Chen L, Stimpson SA, Diehl AM (2006). PPAR*γ* agonists prevent TGF*β*1/Smad3-signaling in human hepatic stellate cells. *Biochemical and Biophysical Research Communications*.

[32] Masamune A, Satoh K, Sakai Y, Yoshida M, Satoh A, Shimosegawa T (2002). Ligands of peroxisome proliferator-activated receptor-*γ* induce apoptosis in AR42J cells. *Pancreas*.

[33] Teresi RE, Shaiu CW, Chen CS, Chatterjee VK, Waite KA, Eng C (2006). Increased PTEN expression due to transcriptional activation of PPAR*γ* by Lovastatin and Rosiglitazone. *International Journal of Cancer*.

[34] Hoshino K, Satoh T, Kawaguchi Y, Kuwana M (2011). Association of hepatocyte growth factor promoter polymorphism with severity of interstitial lung disease in Japanese patients with systemic sclerosis. *Arthritis & Rheumatism*.

